# Evidence of hidden leprosy in a supposedly low endemic area of Brazil

**DOI:** 10.1590/0074-02760170173

**Published:** 2017-12

**Authors:** Fred Bernardes, Natália Aparecida de Paula, Marcel Nani Leite, Thania Loyola Cordeiro Abi-Rached, Sebastian Vernal, Moises Batista da Silva, Josafá Gonçalves Barreto, John Stewart Spencer, Marco Andrey Cipriani Frade

**Affiliations:** 1 Universidade de São Paulo, Faculdade de Medicina de Ribeirão Preto, Departamento de Clínica Médica, Divisão de Dermatologia, Ribeirão Preto, SP, Brasil; 2 Universidade Federal do Pará, Laboratório de Dermato-Imunologia, Marituba, PA, Brasil; 3 Universidade Federal do Pará, Laboratório de Epidemiologia Espacial, Castanhal, PA, Brasil; 4 Colorado State University, Department of Microbiology, Immunology and Pathology, Fort Collins, CO, USA

**Keywords:** leprosy, Mycobacterium leprae, serology

## Abstract

**OBJECTIVES:**

Show that hidden endemic leprosy exists in a municipality of inner São Paulo state (Brazil) with active surveillance actions based on clinical and immunological evaluations.

**METHODS:**

The study sample was composed by people randomly selected by a dermatologist during medical care in the public emergency department and by active surveillance carried out during two days at a mobile clinic. All subjects received a dermato-neurological examination and blood sampling to determine anti-PGL-I antibody titers by enzyme-linked immunosorbent assay (ELISA).

**RESULTS:**

From July to December 2015, 24 new cases of leprosy were diagnosed; all were classified as multibacillary (MB) leprosy, one with severe Lucio's phenomenon. Seventeen (75%) were found with grade-1 or 2 disability at the moment of diagnosis. Anti-PGL-I titer was positive in 31/133 (23.3%) individuals, only 6/24 (25%) were positive in newly diagnosed leprosy cases.

**CONCLUSIONS:**

During the last ten years before this study, the average new case detection rate (NCDR) in this town was 2.62/100,000 population. After our work, the NCDR was raised to 42.8/100,000. These results indicate a very high number of hidden leprosy cases in this supposedly low endemic area of Brazil.

In 2015, although the leprosy detection rate in Brazil reveals a high endemicity pattern with 14.07 cases per 100,000 inhabitants, it is worth noting its geographic heterogeneity, with southern states in which there is low endemicity, such as Rio Grande do Sul (1.08/100,000), and others with high, very high or even hyperendemic, such as Mato Grosso (93.00/100,000). In addition, 19 states, that is around 50% of the population, are exposed to an endemic pattern ranging from high to hyperendemic (from 10 cases/100,000 to > 40 cases/100,000 population) (SINAN 2017).

In São Paulo state, southeastern Brazil, there was a significant drop in leprosy detection coefficients after the introduction of the standardised multidrug therapy (MDT) and, from 2006, this state was considered non-endemic for the disease with a prevalence rate below 1/10,000 inhabitants, reaching 0.23/10,000 in 2015. Parallel to this fact, in the ranking rate of states, São Paulo occupies the third position among those with the lowest overall detection coefficient of new cases (2.73/100,000 inhabitants). Different from many regions in Brazil, all indicators in São Paulo state indicate a tendency to control the disease in most of its municipalities. In 2014, Jardinópolis, in the far north of the state, had a population of 41,228 inhabitants, and had a detection coefficient of new cases of 4.76/100,000 inhabitants and a prevalence rate of 0.73/10,000 inhabitants (IBGE 2017, SINAN 2017), a rate considered medium and low endemics respectively at that time.

According to some authors (Barbieri et al. 2016, Salgado et al. 2016), the true number of leprosy cases in the world in endemic countries is unknown, but is generally thought to be between 6-8 fold higher than the reported number of new cases (Kumar et al. 2007, Moet et al. 2008, Basel et al. 2014). This situation seems to be related to the low capacity of general health workers to perform the diagnosis of leprosy that is based essentially on the identification of clinical signs and symptoms. Currently there is no laboratory test capable of diagnosing all clinical forms of leprosy. Although bacilloscopy is useful in confirming diagnosis in multibacillary (MB) patients and presents a high specificity, it is unable to identify most paucibacillary (PB) patients.

Although the enzyme-linked immunosorbent assay (ELISA) test to detect anti-PGL-I IgM titer is positive in > 95% of all lepromatous patients [borderline lepromatous (BL) and polar lepromatous (LL)], individuals at the tuberculoid end of the spectrum [polar tuberculoid (TT) and borderline tuberculoid (BT)] show only 20-40% positivity or are negative, and care should be given to draw any conclusions about being positive since it is known that 90% of those infected with *Mycobacterium leprae* are naturally immune and will never progress to disease. It has been used as an indicator of contact with *M. leprae* antigens in the general population (Lobato et al. 2011), and it has been show that seropositive household contacts of leprosy patients are three times more likely to develop leprosy when compared to seronegative ones (Barreto et al. 2015, Penna et al. 2016). In addition, IgM anti-PGL-I can be a marker of the intensity of transmission, reflecting the level of endemicity in the community (van Beers et al. 1999).

This article tells a history that started fortuitously in an emergency medical service of a supposedly low endemic town of Brazil (Jardinópolis, São Paulo state). At the end, the collected data reveals a very high hidden prevalence of leprosy in this area based on clinical and immunological findings.

## SUBJECTS AND METHODS

*Ethics, consent and permissions* - This study was approved by the Research Ethics Committee at the Clinics Hospital of Ribeirão Preto Medical School, University of São Paulo (protocol number 16620/2014 HCFMRP-USP). An informed written consent was obtained from every individual who agreed to participate in this study. All procedures involving human subjects comply with the ethical standards of the relevant national and institutional committees on human subjects' experimentation and with the Helsinki Declaration of 1975, as revised in 2008.

*Sampling design and methods* - From July to December 2015, during 24 medical shifts (average of 50 visits/medical shift) performed at the Jardinópolis emergency department (ED) by a dermatologist with experience in leprosy diagnosis, 12 new cases of leprosy were circumstantially detected in 1,200 people evaluated (1.0%) clinically with several clinical complains. Because of this unexpectedly high number of cases initially identified at the ED, a structured campaign to detect new cases in the surrounding community was performed. This included a mobile clinic that was parked from November 24th to 25th, 2015, in a central square of Jardinópolis. The announcement of the campaign was made with a sound advertisement car that went throughout urban neighborhoods during the week prior to the action. Dermatologists, biomedical personnel and physiotherapists from the National Reference Center of Sanitary Dermatology with Leprosy Approach (CRNDSHansen) participated in the action. During this campaign, general health workers from the Jardinópolis municipality were trained to recognise signs and symptoms of skin lesions, loss of sensation, and nerve damage to assist in identifying possible cases, aiming to strength the local leprosy control program.

The enrolled subjects underwent a standardised clinical dermato-neurological examination, as recommended by the World Health Organization (WHO). Leprosy diagnosis was made by the finding of at least one of the following signs/symptoms: (A) definite loss of sensitivity and/or some dysautonomia in a hypochromic or reddish skin macule or (B) a thickened or enlarged peripheral nerve with a respective loss of sensitivity and/or muscle weakness. All leprosy diagnoses were certified by at least two experts. Considering that none of the classifications for leprosy include all of clinical manifestations of leprosy, particularly those involving macular and pure neural forms, we classified the patients considering the guidelines adapted by Ridley-Jopling (Ridley & Jopling 1966), Madrid (Congress of Madrid 1953) and Indian Association of Leprology (IAL 1982) classifications, as follows: indeterminate (I), polar tuberculoid (TT), borderline tuberculoid (BT), borderline borderline (BB), borderline lepromatous (BL), polar lepromatous (LL) and pure neural (N); and according to WHO operational criteria [PB (TT) and MB (BT, BB, BL and LL)]. All newly diagnosed patients were referred to a health unit for standard MDT.

*Serology to detect IgM anti-PGL-I by ELISA* - The titer of anti-PGL-I antibodies in patient and control samples was determined as previously described (Frade et al. 2017). Briefly, ELISA plate wells were coated at 4°C overnight with 12.5 ng synthetic ND-O-BSA in 50 μL of 0.1M carbonate/bicarbonate pH 9.6 coating buffer. Wells were washed and blocked for 1 h with 200 μL 1% bovine serum albumin (BSA) in phosphate buffered saline (PBS), pH 7.2, containing 0.05% Tween20 (blocking solution). Diluted plasma (1:400, 100 μL diluted in blocking solution) was pipetted into duplicate wells and included a blank well coated only with BSA for the negative antigen control, and subsequently incubated for 2 h at room temperature (RT). Then, the wells were washed with PBS plus 0.05% Tween20 (PBS/T, wash buffer) six times. Secondary peroxidase-conjugated anti-human IgM (1:20,000 diluted in PBS/T) was added for another 2 h incubation period. Following this incubation, the wells were washed with PBS/T six times followed by the addition of 100 μL of substrate (3,3', 5,5'-tetramethylbenzidine; TMB, Abcam). After 15 min at an RT-incubation, 50 μL of stop solution (H2SO4, 1 M) was added. Optical density (OD) values were determined with an ELISA plate reader (Asys Expert Plus-Microplate Reader UK) at 450 nm. The respective index was calculated by dividing the OD of each sample by the cut-off, and indexes above 1.0 were considered positive.

*Spatial epidemiology* - The street addresses of all subjects included in this study were georeferenced with a handheld global positioning system (GPS) device (Garmin eTrex H, Olathe, KS, USA) to produce maps of leprosy and subclinical infection distribution in the town. Using QGIS 2.18.3 (http://www.qgis.org), we drew point pattern maps, calculated the number of cases per urban census tract and identified priority areas for additional active surveillance.

## RESULTS

Of the approximately 1,200 people were evaluated during the 24 medical shifts performed by our dermatologist/researcher in the Jardinópolis ED, 12 individuals (1%) were diagnosed with leprosy (one BT, nine BB and two LL), including a case presenting with severe Lucio's phenomenon.

Among 120 people evaluated at the mobile clinic during the two-day campaign, eight (6.4%) new cases were detected (one BT and seven BB). Additionally, after the campaign, the local health professionals that were trained by our team evaluated 78 household contacts of these eight newly diagnosed cases and detected four (5.1%) new leprosy cases (one BT and three BB). Considering all 24 new cases, the age ranged from 14 to 70 years old (average 42.1) and 13 were female (54.1%) (Table).

**TABLE:** Epidemiologic characteristics of the new cases detected

Category		Subcategory	Leprosy cases	
			(n)	(%)
	Gender	Male	11	45.8
		Female	13	54.2
	Age group	< 15 years old	2	8.3
		≥ 15 years old	22	91.7
Place of birth				
		Jardinópolis (SP)	14	58.3
		São Joaquim da Barra (SP)	1	4.2
		Brodowski (SP)	1	4.2
		Other states	8	33.3
Dwelling time in Jardinópolis				
		Average		28.7 years
		1st quartile		15 years
		Median		27 years
		3rd quartile		41 years
	Classification	I/TT/N	0	0
		BT	3	12.5
		BB	19	79.2
		BL	0	0
		LL	2	8.3
	Degree of disability	0	6	25
		1	10	41.7
		2	8	33.3
	BCG scar	0	8	33.3
		1	16	66.7
		≥ 2	0	0
	Anti-PGL-I	Seronegative	18	75
		Seropositive	6	25

SP: São Paulo state; I: indeterminate; TT: tuberculoid leprosy; BT: borderline tuberculoid leprosy; BB: borderline borderline leprosy; BL: borderline lepromatous leprosy; LL: lepromatous leprosy; N: pure neural manifestations.

**Fig. 1:**
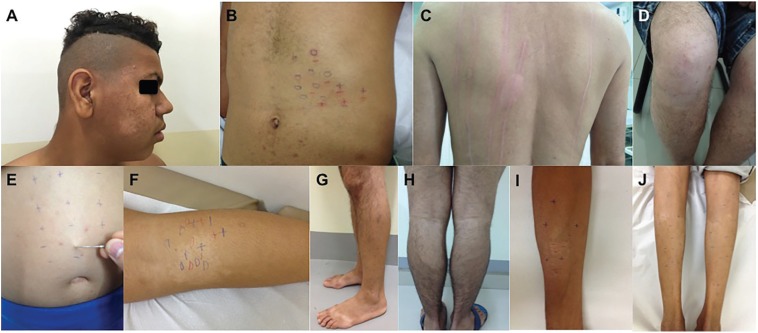
(A) anesthetic hypochromic macule on the right hemiface; (B) points with altered sensitivity (hypoesthetic and anesthetic) inside the area of hypopigmented spot; (C) absence of secondary erythema reflex on leprosy hypochromic lesion as compared to peripheral areas after strong scrawl through the lesion in the endogenous histamine test in hypochromiant dimorphous leprosy patient; (D) anesthetic hypochromic macule with alopecia on the right knee; (E) hypochromic macula with pain anesthesia showed with a needle tip test; (F) hypochromic hypoesthetic and anesthetic macule on the right leg; (G) distal third of the left leg with alopecia, hypoesthesia and anesthesia; (H) hypochromic macule with alopecia on the proximal dorsal half of the left leg; (I) hypochromic macule on the right forearm; (J) multiple hypochromic macules with altered sensitivity, with alopecia, on both legs. Auxiliary clinical tests: “+” normoesthesia (preserved sensitivity); “0” anesthesia (lack of sensitivity); “-” hypoesthesia (perception preserved but less intense than in normoesthetics areas); blue point: tactile sensitivity tested; red point: pain sensitivity tested.

**Fig. 2:**
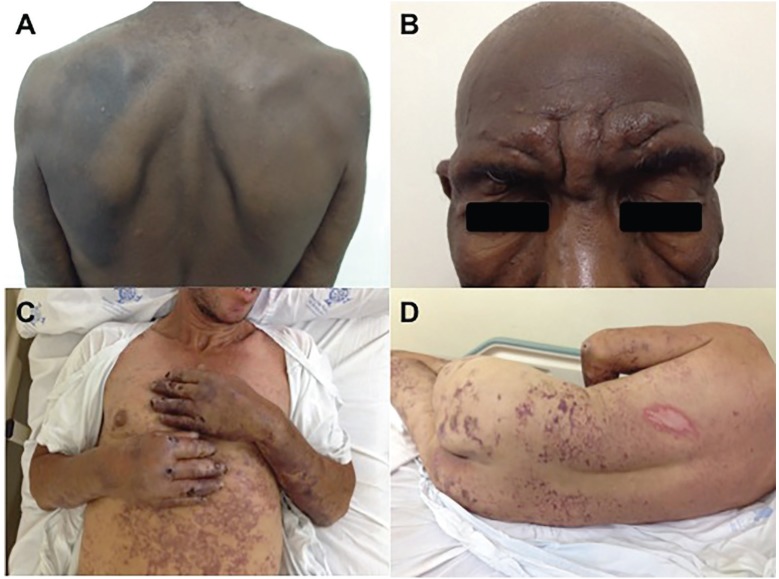
(A, B) lepromatous patient with multiple papules and nodules on back and face; (C, D) patient with Lucio's phenomenon - necrotising lesions over the legs, buttocks and dorsum.

The diagnosis was established with hypochromic macules with alteration of sensitivity (tactile and/or thermal and/or painful) in 20 patients (Fig. 1), two individuals presented a xerotic plaque with anesthesia in islet (one on the right foot and one on the right hand, the latter with interosseous hypoatrophy). Two classic lepromatous patients, both with diffuse infiltration and madarosis; one with multiple anesthetic papules and nodules and the other with dark, irregular-shaped, bizarre, erythematous, purpuric spots and ulcers on upper, lower limbs and trunk including dorsum of hands, finger tips, ears and tongue (Fig. 2). In 19 individuals in whom endogenous histamine was tested, it was incomplete; ten presented alopecia areas; the most commonly affected neural trunks were: common fibular (11/24, 45.8%), posterior tibial (10/24, 41.2%), ulnar (7/24, 29.2%), median and supraorbital (1 each/4.2%). Topographically, the lesions were distributed in the following areas: lower limbs (11/24, 45.8%), dorsum (8/24, 33.3%), upper limbs (7/24, 29.2%), face, back and trunk (two each/8.3%). Eighteen (75%) patients had some physical disability (GD), ten with G1D (41.7%) and eight with G2D (33.3%).

Only among patients and individuals evaluated during the campaign, 133 blood samples were collected and anti-PGL-I was positive in 31 (23.3%) individuals, although only four (0.3%) had a known history of contact with leprosy. Among the 24 patients, only six (25%) were positive, two BB and two LL. Nonetheless, 12 subjects with no clinical signs or symptoms of leprosy displayed an ELISA index to IgM anti-PGL-I over 2.0, that is comparable to most MB cases with a high bacillary index (BI), indicating a higher risk of leprosy in the future.

Fourteen (58.3%) patients were born and raised in Jardinópolis, two others (8.4%) were also born in municipalities of São Paulo state and eight in other states. When analyzing the dwelling time in Jardinópolis, the average (28.7 years) and the median (27 years) had close values. The value of the first quartile was 15 years and the third quartile was 41 years of residence in Jardinópolis.

**Fig. 3:**
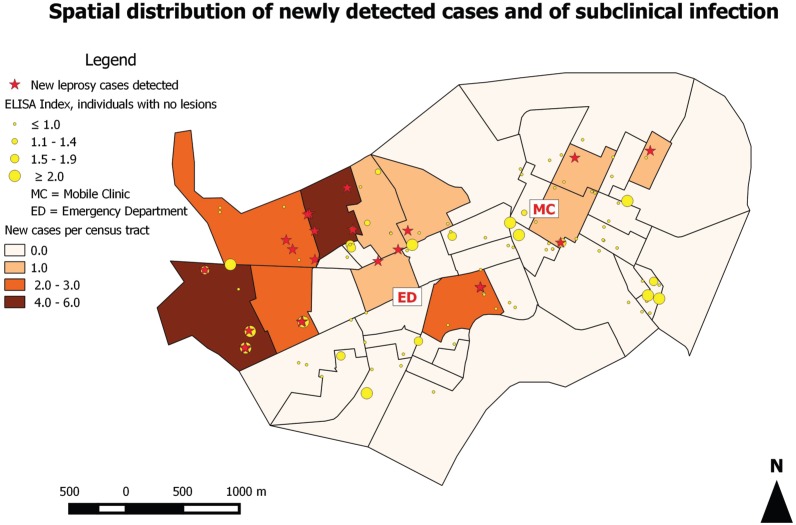
spatial distribution of newly detected cases and of subclinical infection. A hotspot of new cases was localised within the northwestern census tracts, accounting for 19 out of 24 new cases detected (79.16%). Additionally, 12 subjects with no clinical signs or symptoms of leprosy displayed Enzime-linked immunosorbent assay (ELISA) indexes to IgM anti-PGL-I over 2.0, that is comparable to multibacillary (MB) cases exhibiting a high bacillary index, indicating higher risk of leprosy in the future.

Of the 112 people evaluated at the mobile clinic that did not exhibit any clinical manifestation of leprosy, we georeferenced 106 (94.6%). Six street addresses were not mapped (two from the rural area, two from another city, one from another state and one lacking address information). Fig. 3 shows the spatial distribution of the newly detected cases, and the subjects without leprosy considering their IgM anti-PGL-I index. It was possible to notice a hotspot of new cases in the northwestern census tracts of Jardinópolis, accounting for 19 out of 24 new cases detected (79.2%).

## DISCUSSION

Although the data presented by the Brazilian Ministry of Health shows that the incidence of leprosy in Brazil is slowly decreasing, the goal of eliminating the disease as a public health problem has not been reached (Salgado et al. 2016). In many cases, the changes for leprosy diagnosis can be subtle and are often a challenge even for specialists. Frade et al. (2017) showed an unexpected high percentage of leprosy cases and high number of individuals with positive serological responses to *M. leprae* antigens in a Brasilia (Federal District, Brazil) population, previously considered a non-endemic area, and suggested faults in the health care delivery system and a lack of training by physicians working in the basic health units as causal factors.

The decentralisation of health care to leprosy patients was accompanied by an attempt to simplify its diagnosis by the WHO based mainly on counting the number of skin lesions. These operative changes can be considered among those responsible for the general decline in the training and expertise of health care professions to recognize clinical signs and symptoms of leprosy, particularly at the early stages of disease, often resulting in misdiagnosis or long delays in diagnosis and treatment even in advanced cases with obvious symptoms of disease (Henry et al. 2016). Consequently, numerous cases that remain undiagnosed and untreated remain, and these individuals then serve as continuing reservoirs of infection and have a profound impact on the maintenance of the chain of transmission.

An unfavorable reality in medical care in several Brazilian municipalities is the turnover of professionals in the primary health care system (Campos & Pereira Jr 2016). Such rotation combined with insufficient medical academic training on leprosy have reduced the effectiveness of training courses offered by municipal and state health departments (Saunderson 2013, Alves et al. 2014, Costa et al. 2015). Different from other regions, the municipality of Jardinópolis has six permanent primary health care teams established there for more than five years.

During the second half of 2015, 51,206 medical appointments were performed at the Jardinópolis ED, with an average of 284 visits/day (unpublished observations). All 12 patients diagnosed at the ED requested medical consulting with complaints not related to leprosy, such as headache, cough, hives, diarrhea, mechanical low back pain, among other issues. In the literature, studies describing leprosy and emergency care are usually related to acute reactional conditions (Hoffner et al. 2000, Nishioka 2001), and there are no studies that describe the detection of new cases at an ED. The presence of a dermatologist in an emergency room can be considered a bias, however, the constant high number of low complexity visits and the diagnosis of classic cases of leprosy in the ED revealed an unsuitable quality of care offered by the family health strategy (FHS). The prevalence of leprosy in Jardinópolis in 2015 deserves to be highlighted as the highest in the state of São Paulo (4.4/10,000), thus now considered in the medium endemic range.

Leprosy is strongly related to poverty. The knowledge about risk areas for leprosy reveals that the distribution of leprosy is intimately linked to a number of factors that coincide to maintaining high rates of transmission and new case detection rate, including environmental, individual, socioeconomic and health service organization factors (Ramos et al. 2017). The spatial distribution pattern of the newly detected cases revealed a hotspot localised within the poorest area of the city. This data could support targeted interventions in this priority area, including door-to-door active clinical surveillance instead of the current passive system, waiting for people with long-term nerve damage and disability to arrive at the basic health unit for an uncertain diagnosis.

Data from the National Notifiable Diseases Information System (SINAN 2017) shows that from 2005 to 2014, only 11 new cases of leprosy were detected in Jardinópolis and no new case was detected during the first semester of 2015. Considering the relatively high number of new cases readily detected in this study, together with the high seroprevalence of IgM anti-PGL-I in the community (stimulated demand), it becomes clear that a high hidden prevalence of leprosy exists in this town. Similarly survey and results were obtained by Frade et al. (2017) in Federal District, another official non-endemic area in Brazil. Surprisingly, our results showed an ELISA index to IgM anti-PGL-I over 2.0 in 10.7% of healthy individuals, higher than 5.9% found in Federal District.

Whereas the majority of patients were born and raised in Jardinópolis as well as the dwelling time in Jardinópolis much longer than the incubation time of leprosy, it becomes unquestionable that the endemic is local. In addition, we highlight the finding of advanced cases such as classic lepromatous leprosy with severe Lucio's phenomenon and the high number of cases diagnosed with established physical disability, indicating a long delay for case detection, diagnosis and treatment. The primary care system of Jardinópolis seems to be lacking in the ability to diagnose leprosy, even in the classic forms. It is evident that the need for continuous active surveillance in this area will be necessary, including strategies for strengthening the local leprosy control program and rebuilding professional expertise in order to achieve a successful reduction from the current hyperendemic detection rate of leprosy in this region.

Considering the results of our study, we highlighted four recommendations in public health: the Ministry of Health must intensify leprosy control activities even in low endemic regions, since this study identified this very high hidden prevalence; general health workers should be continuously trained to identify early signs and symptoms of leprosy; we strongly recommend door-to-door active surveillance, particularly in priority neighborhoods, based on the spatial distribution of the cases; the community should be aware of the clinical manifestations of leprosy in hyperendemic areas. In this way, all kinds of media (TV, radio, internet, social media, etc.) have a key role in spreading information to inform the general public, not only to the health workers.
